# Prevalence and 9‐year incidence of hepatitis E virus infection among North Italian blood donors: Estimated transfusion risk

**DOI:** 10.1111/jvh.13296

**Published:** 2020-04-08

**Authors:** Marta Spreafico, Livia Raffaele, Irene Guarnori, Barbara Foglieni, Alessandra Berzuini, Luca Valenti, Alessandro Gerosa, Agostino Colli, Daniele Prati

**Affiliations:** ^1^ Department of Transfusion Medicine and Hematology Alessandro Manzoni Hospital, ASST‐Lecco Lecco Italy; ^2^ Department of Transfusion Medicine and Hematology Fondazione IRCCS Cà Granda Ospedale Maggiore Policlinico Milan Italy; ^3^ Department of Pathophysiology and Transplantation Università degli Studi di Milano Milan Italy; ^4^ Department of Internal Medicine Alessandro Manzoni Hospital, ASST‐Lecco Lecco Italy

**Keywords:** hepatitis E virus, incidence, prevalence, transfusion transmission

**Abbreviations**
CIs: confidence intervals
HEV: hepatitis E virus
ID‐NAT: individual nucleic acid testing
NAT: nucleic acid testing

## 1 INTRODUCTION

Hepatitis E virus (HEV) is mainly spread in humans by contaminated food and water, but it is increasingly being recognized as a threat to blood transfusion safety because of its documented transmission by means of viremic blood components.^1^, ^2^

The risk of transfusion‐related infection is generally estimated on the basis of the prevalence of HEV RNA among blood donors. Nucleic acid testing (NAT) has detected a high rate of viremic donations (up to one in 600) in a number of European countries.^1^, ^2^ There is some evidence of high prevalence of viremia and anti‐HEV reactivity among donors in Abruzzo (Central Italy),^3^ although a retrospective analysis conducted in plasma pools by the Italian National Blood Centre suggests that the pattern of HEV circulation might be different in other Italian regions.^4^

However, pooling procedures can limit the analytical sensitivity of NAT and so donations should undergo individual testing. Furthermore, a number of reports indicate that the infection pressure is not stable over time. This implies that NAT yields determined in the relatively short time frame of a prevalence study may not be entirely representative of the risk of transfusion‐related transmission, and serological incidence data may be more useful.

Taking advantage of a longitudinal biorepository financed by the European Union,^5^ we calculated the prevalence and incidence of HEV infection over the last ten years in donors from Northern Italy and used these data to estimate the risk of the transfusion‐related transmission of HEV infection.

## 2 SUBJECTS AND METHODS

The study was conducted within the framework of the EU‐funded Blood and Organ Transmissible Infectious Agents (BOTIA) project (SP23‐CT‐2006‐006487).^5^ The study protocol was approved by our local Institutional Review Board and conducted in accordance with Italian Authorisation No. 9/2014 of 11 December 2014 concerning personal data protection for scientific research purposes. The donors underwent biochemical and virological testing as prescribed by Italian regulations.

Frozen plasma samples from donations collected at the Department of Transfusion Medicine and Hematology in Lecco in 2015‐2016 were tested for the presence of circulating HEV RNA by means of individual NAT (ID‐NAT), using the Procleix HEV assay and Panther instruments (Grifols, Barcelona, Spain), with a 95% limit of detection of 7.9 IU/mL. Donors found to be initially ID‐NAT reactive were re‐tested on a different aliquot, tested for anti‐HEV IgG and IgM (DiaPro HEV IgG and HEV IgM kits; Diagnostic BioprobesSrl) and followed up at subsequent donations.

A subset of samples was also analysed for the presence of anti‐HEV IgG in order to study the prevalence of past exposure to HEV. Confirmation of initially reactive samples was based on repeated testing and, when possible, on follow‐up.

Finally, in order to evaluate the dynamics of HEV infection over time, we identified a subgroup of donors who had given two serial samples over a relatively long follow‐up: that is the first at the start of the BOTIA project in 2007‐2010, and the second in 2017. These samples were tested for anti‐HEV IgG, and the incidence of infection was calculated as the number of seroconversions by the total number of initially negative cases, multiplied by the median number of years of follow‐up and expressed as a number per 10 000 per year. The risk of receiving an infectious blood unit was estimated using two methods: HEV RNA yield and serological incidence, assuming a viremia duration of four weeks in the case of asymptomatic infections.^6^

## 3 RESULTS

**FIGURE 1:**
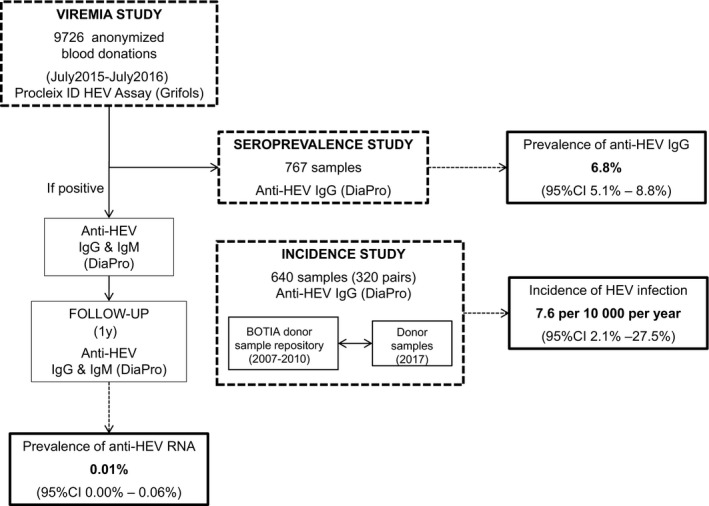
Flow chart and main results of the study

Figure 1 summarizes the study results.

A total of 9726 donor samples were collected for the HEV RNA study: 7253 (74.5%) taken from males and 2473 (25.5%) taken from females, with a mean age of 43 years (range: 18‐67).

The ID‐NAT assay showed that ten of the samples were initially reactive, but repeated testing confirmed reactivity in only one. The donor was a 63‐year‐old female who had normal alanine aminostransferase levels (ie 22U/L) and was fully asymptomatic at the pre‐donation examination. She subsequently seroconverted to being anti‐HEV IgG positive. None of the nine donors whose initial reactivity was not confirmed seroconverted during the follow‐up period. Thus, the prevalence of HEV RNA was 0.01% (95% CI 0.00%‐0.06%).

In addition, 767 samples (76.7% males, 23.3% females, mean age 43 years) were analysed in order to determine the prevalence of anti‐HEV. Anti‐HEV IgG reactivity was confirmed in fifty‐two donors (45 males and 7 females), thus indicating an overall prevalence of IgG of 6.8% (95% CI 5.1%‐8.8%). None of them was concomitantly anti‐HEV IgM or HEV RNA positive. The prevalence of anti‐HEV IgG increased across age strata, ranging from 1% (95% CI 0.0%‐5.7%) in donors of 18‐30 years to 11.1% (95% CI,7%‐16.5%) in those of 50 years or older.

Finally, a subset of 320 donors contributed 640 paired samples collected between 2007 and 2017 (mean interval between sampling: 9 years, range 8‐10). Thirty‐one donors (9.7%; 95% CI: 6.7‐13.5%) were anti‐HEV IgG positive at baseline, and all of them were still reactive when the second sample was collected. Two of the 289 donors who were initially anti‐HEV‐IgG negative had seroconverted by the time of the follow‐up sample: they were both males and, in 2017, were, respectively, 48 and 49 years old. The incidence was 7.6 (95% CI 2.1‐27.5) per 10 000 per year. The estimated risk of transfusion‐related infection based on HEV RNA yields was 1/10000 blood donations (the upper limit of the 95% CI was 1/1,666); the estimate based on the incidence data was 1/16666 blood donations (95% CI 1/4350‐1/57000).

## 4 DISCUSSION

Our study combined HEV viremia and serological data in order to estimate transfusion risk over a ten year period. Using high sensitive individual testing, we found that only one of the ten initially HEV RNA reactive samples in our series was found to be truly viremic, accounting for one out of almost ten thousand blood donations. In addition, the overall seroprevalence of anti‐HEV IgG was 6.8%. These data indicate that the frequency of current and past HEV infections among blood donors in northern Italy is one of the lowest so far reported in Europe. According to recent reviews, the prevalence of HEV RNA positivity ranges from 1/762 in the Netherlands to 1/8416 in Austria and that of anti‐HEV ranges from 12% in England to 53% in south‐western France.^2^ Our findings are also very different from those observed in Abruzzo, a region in central Italy. According to Lucarelli et al ^3^, almost half of the subjects donating blood in L’Aquila during the first months of 2014 showed serological signs of previous HEV exposure, and one out of 166 had detectable viremia. The highly endemic nature of the infection in this area has been attributed to local dietary habits favouring zoonotic transmissions, but another possibility is contaminated water, as the circulation of some faecal pathogens increased in the area of L’Aquila for several years after the catastrophic earthquake in 2006.^7^

Given that nine of the ten initially NAT‐reactive samples were not confirmed by further testing or at serological follow‐up, it seems that a single determination of HEV RNA has little positive predictive value. The impact of false‐positive results might be more evident when testing samples at low risk of infection, like blood donors. It is therefore possible that some of the variability in the prevalence rates recorded in previous studies is related to methodological differences.

As expected, the prevalence of anti‐HEV increased with age, thus reflecting a cohort effect and the long‐term persistence of antibody reactivity after primary infection. The fact that the age‐related increases in prevalence were relatively uniform (data not shown) suggests that no major outbreak has occurred since the 1950s. Our prospective repository of biological samples allowed us to calculate the incidence of HEV infection over a ten year period, which proved to be 7.6/10 000 per year and is substantially lower than the calculated incidence in other European countries such as Germany (35/10 000 per year).^8^ Furthermore, these data allowed us to estimate the local transfusion‐related risk of infection using a method based on the assumption that the viremic phase (and therefore, the infectiousness of the blood products collected during active infection) lasted four weeks in each seroconverting blood donor.^6^ On the basis of this calculation, approximately one out of 16 000 donors should be positive at any given time, a figure that is in line with the prevalence of HEV RNA found in this study and confirms that infection pressure remained quite stable in northern Italy during the considered ten years.

The limitations of this study include the fact that we could not look back on HEV transmission because all of the samples were coded and blinded to investigators, according to current regulations. Secondly, the findings cannot be extended to the Italian population as a whole because of the geographical heterogeneity of the circulation of HEV. However, our study population was representative of the local community of blood donors as it included donors living in urban, suburban, rural and mountain areas. Lombardy is the most highly populated region in Italy, and its transfusion system provides 24% of the total Italian blood supply. Although the circulation of HEV is minimal in comparison with other European countries, our data indicate that some tens to hundreds of HEV‐infected blood components a year may be transfused into blood recipients. Some of them, such as immunocompromised patients, are susceptible to the development of acute or chronic liver failure, or a chronic infection that may rapidly progress to liver cirrhosis and death.^1^, ^2^

The findings of this study may be useful for the regional and national blood authorities responsible for making policy decisions given that recent European guidelines^1^ recommend that HEV screening policies should be based on local risk assessment studies. Whether or not to introduce HEV NAT screening therefore requires careful consideration: donor screening may very effectively minimize iatrogenic HEV infection, but it is very costly and can be expected to have a relatively minor impact on the number of HEV infections in the population as a whole because the vast majority of new infections seems to be due to dietary exposure.^1^, ^2^

## 5 CONFLICT OF INTEREST

Grifols Italia S.p.A (Milan, Italy) and Diagnostic BioprobesSrl, (Milan, Italy) provided the kits for serological and molecular analyses.
